# Travelers’ vaccines and their adverse events in Nara, Japan

**DOI:** 10.1515/med-2021-0303

**Published:** 2021-06-30

**Authors:** Taku Ogawa, Nobuyasu Hirai, Natsuko Imakita, Hiroyuki Fujikura, Akihiro Kajita, Yuichiro Imai, Tomoko Onishi, Masahiro Takeyama, Kei Kasahara

**Affiliations:** Center for Infectious Diseases, Nara Medical University, 840 Shijo-Cho Kashihara, Nara 634-8522, Japan; Department of Pathogen, Infection and Immunity, Nara Medical University, Nara, Japan; Department of Pediatrics, Nara Medical University, Nara, Japan

**Keywords:** typhoid vaccine, rabies vaccine, hepatitis A vaccine, cost payer, unapproved vaccine

## Abstract

**Background:**

It is important to analyze the types of vaccines in travel clinics to determine the focus points in future practice.

**Methods:**

We retrospectively reviewed the electronic medical records of all patients who visited the travel clinic of Nara Medical University between June 2013 and December 2019 to determine their background and the vaccines administered. The information regarding adverse events of the unapproved vaccines in Japan (Havrix^®^, Verorab^®^, Boostrix^®^, Priorix^®^, Typhim Vi^®^, and Mencevax^®^) was also collected.

**Results:**

Of 645 patients, 58.6% were men and the median age was 31 years. Business was the most common travel purpose (34.9%), and Southeast Asia was the most common destination (40.2%). More than 80% of travelers to low- and middle-income countries were vaccinated against hepatitis A, while the rabies vaccination rate was approximately 50%. Typhoid vaccination coverage among travelers to South Asia was approximately 50%. The incidence of adverse events requiring medical consultation, telephonic consultation, or prolonged stay in the examination room was less than 5% for all unapproved vaccines in Japan.

**Conclusion:**

More patient education is needed to increase the vaccination rate of rabies and typhoid vaccines. Adverse events to unapproved vaccines in Japan were not high and were well-tolerated.

## 1 Introduction

Until the global pandemic of coronavirus disease, which originated in Wuhan in 2019, the number of international travelers from Japan continued to increase, with the number of international travelers exceeding 20 million in 2019. Compared to other developed countries, the practice of visiting travel clinics for pretravel health consultations, including vaccinations, was less widespread in Japan. To improve this situation, Nara Medical University opened a travel clinic in June 2013 and has been operating it following the center for disease control and prevention and the Japanese society of travel health recommendations [1,2]. We believe that it is important to analyze the content of the vaccines and other medical care provided to our clients for better care in the future. The data will also be of interest to doctors, nurses, and other healthcare professionals who run other travel clinics. For the aforementioned reasons, we compiled a list of the characteristics of clients who visited our travel clinic, the areas they traveled to, and vaccines and their forms that were finally administered. We also analyzed the extent to which vaccine adverse events occurred and how factors such as who paid for the visit affected the vaccines administered.

## 2 Materials and methods

The Nara Medical University Center for Infectious Diseases established a travel clinic in June 2013. The travel clinic is open on Monday and Thursday afternoons by appointment only, and the available vaccines are listed in Table 1; all vaccines are kept in stock at the pharmaceutical department of our hospital for immediate use. Vaccines not approved for manufacture in Japan (Havrix^®^, Typhim Vi^®^, Mencevax^®^, Verorab^®^, Priorix^®^, and Boostrix^®^) were imported individually with the approval of the Nara Medical University Ethics Committee and used after obtaining patient consent. We retrospectively reviewed the medical records of all medical clients who had an initial consultation between June 2013 and December 2019 to determine age, sex, nationality, region of residence, occupation, destination, the purpose of travel, cost payer, planned duration of travel, whether a medical certificate was required, post-vaccination adverse events, and information on the type of vaccine given and prophylactic oral medications administered and the adverse events (beyond the extended stay in the examination room or the need for telephonic consultation). Destinations were categorized by geographic region as well as by the income level of the country. The country’s income level was classified into three categories according to the annual gross national income (GNI) per capita in 2017: high-income countries (≥12,746 USD), middle-income countries (1046–12,745 USD), and low-income countries (≤1045 USD). When visiting more than one country, the country with the lowest GNI per capita was considered the destination. We then tabulated the vaccines administered in each destination region. To examine the effect of cost-sharing on vaccination rates, we used Fisher’s exact probability test to determine whether there were differences in vaccination rates depending on whether or not people had to pay for the vaccine themselves. Specifically, we examined whether there was a difference in the proportion of recipients whose costs were paid for by others, including their companies or schools, for hepatitis A, rabies, and typhoid vaccines among travelers to low- and middle-income countries in the vaccination and non-vaccination groups. Calculations were performed using the Microsoft Excel 2019 software. All *P*-values were two-sided, and *P*-values ≤ 0.05 were considered to be statistically significant. For adverse events as this was a retrospective study, we were only able to count adverse events with prolonged stay in the examination room just after inoculation of vaccines and with a telephonic or face-to-face consultation. The use of unauthorized vaccines in Japan and in this study was conducted with the approval of the Ethical Review Committee of Nara Medical University Ethics Committee (approval number 660).

**Table 1:** Vaccines available at Nara Medical University Travel Clinic

Types of vaccines		Product name	Manufacturing approval in Japan
Hepatits A vaccine		Aimmugen	〇
		Havrix	✖
Hepatitis B vaccine		Bimmugen	〇
		Heptavax-II	〇
Rabies vaccine (JRV)		Inactivated tissue culture rabies vaccine	〇
	(PVRV)	Verorab	✖
	(PCEC)	Rabipur	〇
Japanese encephalitis vaccine (JE)		JEBIK-V	〇
Diphteria, tetanus, pertussis, poliomyelitis vaccine (DTaP)		Quattorovac	〇
Diphteria, tetanus, pertussis vaccine (Tdap)		Boostrix	✖
Tetanus toxoid (TT)		ADSORBED TETANUS TOXOID	〇
Inactivated poliomyelitis vaccine (IPV)		IMOVAX POLIO subcutaneous	〇
Measles, Rubella, Mumps combined vaccine (MMR)		Priorix	✖
Japanese bivalent MR combined vaccine (bMR)		FREEZE-DRIED LIVE ATTENUATED MEASLES AND RUBELLA COMBINED VACCINE	〇
Japanese monovalent measles vaccine (mMe)		DRIED LIVE ATTENUATED MEASLES VACCINE	〇
Japanese monovalent rubella vaccine (mR)		DRIED LIVE ATTENUATED RUBELLA VACCINE	〇
Japanese monovalent mumps vaccine (mMu)		DRIED LIVE ATTENUATED MUMPS VACCINE	〇
Japanese monovalent varicella vaccine (mV)		VARICELLA VACCINE LIVE ATTENUATED “BIKEN”	〇
Meningococcal vaccine (MCV4)		Menactra	〇
	(MPSV4)	Mencevax	✖
Typhoid fever (ViCPS)		Typhim Vi	✖
Pneumococcal vaccine (PCV13)		Prevenar 13	〇
	(PPSV23)	Pneumovax NP	〇
Haemophilus influenaze b vaccine		ActHIB	〇
Influenza virus vaccine (IIV4)		Influenza HA vaccine “SEIKEN”	〇

Tdap, tetanus and reduced diphtheria toxoids and acellular pertussis vaccine for adults; IIV4, inactivated quadrivalent influenza vaccine; MR, Measles-rubella combined; JRV, Japanese rabies vaccine; PVRV, purified vero cell rabies vaccines; PCEC, primary chick embryo cell vaccine; MCV4, quadrivalent conjugate meningococcal vaccine; MPSV4, quadrivalent polysaccharide meningococcal vaccine; PCV13, 13-valent pneumococcal conjugate vaccine; PPSV23, 23-valent pneumococcal polysaccharide vaccine; ViCPS, Vi capsular polysaccharide vaccine.

## 3 Results

### 3.1 Characteristics of clients who visited our travel clinic

A total of 645 patients visited the Nara Medical University Travel Clinic between June 2013 and December 2019. Of whom, 378 (58.6%) were men and 267 (41.4%) were women. The median age was 31 years, the youngest was 0 years old, the oldest was 82 years old, and the interquartile range (IQR) ranged from 19 to 40 years. The breakdown of nationalities was 636 Japanese, two Vietnamese, one Malaysian, and five unknowns. The most common purpose of travel was tourism for 96 (14.88%), followed by the business for 225 (34.9%), accompanying the family to business travelers for 174 (27.0%), studying abroad for 107 (16.6%), volunteering for 24 (3.72%), visiting friends and relatives for 10 (1.55%), participation in sports events for five (0.8%), and participation in religious events for four (0.6%). The breakdown of travel duration was 135 (20.9%) planned to travel for 2 weeks or less, 161 (24.9%) for longer than 2 weeks and up to 1 year, and 349 (54.1%) for longer than 1 year.

Southeast Asia was the most popular destination with 259 (40.2%) individuals, followed by South Asia with 109 (16.9%), North America with 90 (14.0%), East Asia with 71 (11.0%), Africa with 29 (4.5%), Latin America with 24 (3.72%), Oceania with 20 (3.1%), West Asia with 11 (1.71%), Central Asia with four (0.62%), and South America with three (0.47%) individuals. Three (0.47%) individuals were planning a round-the-world trip, and one (0.2%) had not yet decided on a destination. A hundred and seventy (26.4%) respondents traveled to high-income countries, 206 (31.9%) to middle-income countries, and 269 (41.71%) to low-income countries (travelers who had not yet decided on a destination and were planning to travel to low-income countries).

### 3.2 Vaccination rate by destination and type of vaccine

Hepatitis A vaccine (including both Japanese hepatitis A vaccine and Havrix^®^) coverage was highest among travelers to Southeast Asia, East Asia, South Asia, Central Asia, and Latin America (>80%). More than 80% of low- and middle-income countries had been vaccinated against hepatitis A. The percentage of travelers who received the hepatitis B vaccine was highest among travelers to East Asia (47.9%); however, it did not reach 50%. The overall hepatitis B vaccination rate was 29.6%.

Rabies (including both Japanese rabies vaccine and Verorab®) vaccination rates exceeded 50% among travelers to Southeast Asia, South Asia, East Asia, West Asia, and Central Asia; however, the overall vaccination rate was 45.1%. Typhoid vaccine coverage was 50.5% among travelers in South Asia, followed by West Asia (36.4%) and Africa (31.0%). Vaccination rates for measles and rubella-containing vaccines were highest in Europe (32.4 and 23.5%, respectively), followed by Southeast Asia (17.0 and 14.7%, respectively) and South Asia (22.9 and 20.2%, respectively). The overall vaccination coverage was 17.1 and 15.2%, respectively. Pertussis-containing vaccine coverage was relatively high in travelers to Oceania (40.0%) and North America (35.6%) and was higher in travelers to high-income countries (29.4%) than in low-income countries (13.8%). Tables 2–5 show the results of vaccines administered and preventive medications prescribed by the destination.

**Table 2:** Sex, age, nationality, and travel duration of travelers who visited the Nara Medical University Travel Clinic

Age (years)	Minimum	0.5	
	Maximum	82	
	Median	31	
Sex	Male	378	58.6%
	Female	267	41.4%
Nationality	Japan	636	98.6%
	Vietnam	2	0.3%
	Malaysia	1	0.1%
	Pakistan	1	0.1%
	Unknown	5	0.8%
Travel duration	<2 weeks	135	20.9%
	≥2 weeks, <1 year	161	25.0%
	≥1 year	349	54.1%

**Table 3:** Travel destinations for persons vaccinated against hepatitis A, rabies, hepatitis B, and typhoid

All clients			Hepatits A				Rabies				Hepatits B		Typhoid fever	
Destination			Aimmu-gen	Havrix	Total		JRV or Rabipur	Verorab	Total		Bimugen or Heptavax-II		Typhim Vi	
	*n*	%	*n*	*n*	*n*	%	*n*	*n*	*n*	%	*n*	%	*n*	%
Southeast Asia	259	40.2	33	192	225	86.9	21	131	152	58.7	79	30.5	38	14.7
East Asia	71	11	25	34	59	83.1	6	35	41	57.7	34	47.9	2	2.8
South Asia	109	16.9	23	65	88	80.7	7	53	60	55	30	27.5	55	50.5
West Asia	11	1.7	1	4	5	45.5	1	5	6	54.5	3	27.3	4	36.4
Central Asia	4	0.6	1	3	4	100	0	4	4	100	1	25	1	25
Africa	29	4.5	8	15	23	79.3	1	7	8	27.6	9	31	9	31
Europe	34	5.3	2	16	18	52.9	1	1	2	5.9	2	5.9	3	8.8
North America	90	14	4	12	16	17.8	1	6	7	7.8	27	30	1	1.1
Latin America	24	3.7	6	14	20	83.3	2	8	10	41.7	8	33.3	3	12.5
Oceania	20	3.1	1	5	6	30	0	3	3	15	2	10	3	15
Round-the-world trip	3	0.5	1	1	2	66.7	0	3	3	100	1	33.3	0	0
Undecided	1	0.2	0	0	0	0	0	0	0	0	0	0	0	0
High-income countries	170	26.4	8	48	56	32.9	2	23	25	14.7	40	23.5	6	3.5
Middle-income countries	206	31.9	46	129	175	85	15	103	118	57.3	80	38.8	19	9.2
Low-income countries	269	41.7	49	176	225	83.6	25	123	148	55	71	26.4	92	34.2
Total	645	100	103	353	456	70.7	42	249	291	45.1	191	29.6	117	18.1

JRV, Japanese rabies vaccine (inactivated tissue culture rabies vaccine).

**Table 4:** Travel destinations for tetanus, pertussis, polio, and meningococcal vaccine recipients

All clients			Tetanus, pertussis, diphtheria, poliomyelitis								Meningococcal			
Destination			Tetanus toxoid	Japanese DTaP-IPV	Japanese DTaP	Boostrix	Tetanus-containing total		pertussis-containing total		Mencevax	Menactra	Total	
	*n*	%	*n*	*n*	*n*	*n*	*n*	%	*n*	%	*n*	*n*	*n*	%
Southeast Asia	259	40.2	62	4	1	33	100	38.6	38	14.7	0	5	5	1.9
East Asia	71	11	17	1	0	19	37	52.1	20	28.2	0	0	0	0
South Asia	109	16.9	29	3	0	10	42	38.5	13	11.9	0	1	1	0.9
West Asia	11	1.7	2	0	0	2	4	36.4	2	18.2	0	3	3	27.3
Central Asia	4	0.6	1	1	0	1	3	75	2	50	0	1	1	25
Africa	29	4.5	10	3	0	2	15	51.7	5	17.2	1	2	3	10.3
Europe	34	5.3	4	1	0	4	9	26.5	5	14.7	1	1	2	5.9
North America	90	14	8	3	0	29	40	44.4	32	35.6	7	20	27	30
Latin America	24	3.7	9	2	0	1	12	50	3	12.5	1	0	1	4.2
Oceania	20	3.1	2	1	0	7	10	50	8	40	0	0	0	0
Round-the-world trip	3	0.5	0	0	1	0	1	33.3	0	0	0	0	0	0
Undecided	1	0.2	0	0	0	0	0	0	0	0	1	0	0	100
High-income countries	170	26.4	17	4	1	45	67	39.4	50	29.4	8	25	33	19.4
Middle-income countries	206	31.9	54	5	0	36	95	46.1	41	19.9	1	4	5	2.4
Low-income countries	269	41.7	67	8	1	28	104	38.7	37	13.8	2	4	6	2.2
Total	645	100	138	17	2	109	266	41.2	128	19.8	11	33	44	6.8

DTaP-IPV, diphtheria-tetanus-pertussis-poliomyelitis combined vaccine; DTaP, diphtheria-tetanus-pertussis combined vaccine.

**Table 5:** Travel destinations for persons vaccinated against measles, rubella, mumps, varicella, and Japanese encephalitis

All clients			Measles, rubella, varicella, mumps										Japanese encephalitis	
Destinations			Priorix	bMR	mMe	mRu	mMu	mV	Measles total		Rubella total		JEBIK-V	
	*n*	%	*n*	*n*	*n*	*n*	*n*	*n*	*n*	%	*n*	%	*n*	%
Southeast Asia	259	40.2	22	11	11	3	8	5	44	17	38	14.7	91	35.1
East Asia	71	11	10	1	1	0	6	1	12	16.9	11	15.5	31	43.7
South Asia	109	16.9	13	8	4	1	4	1	25	22.9	22	20.2	31	28.4
West Asia	11	1.7	0	2	0	1	1	0	2	18.2	2	18.2	2	18.2
Central Asia	4	0.6	0	0	0	0	1	0	0	0	0	0	1	25
Africa	29	4.5	0	0	0	0	0	0	0	0	0	0	2	6.9
Europe	34	5.3	5	2	2	0	2	0	11	32.4	8	23.5	0	0
North America	90	14	5	6	1	1	25	12	12	13.3	12	13.3	2	2.2
Latin America	24	3.7	2	0	0	0	0	1	2	8.3	0	0	2	8.3
Oceania	20	3.1	4	0	0	1	2	0	4	20	4	20	2	10
Roud-the-world trip	3	0.5	0	0	0	0	0	0	0	0	0	0	0	0
Undecided	1	0.2	0	0	0	0	0	0	0	0	0	0	0	0
High-income countries	170	26.4	15	10	5	1	30	12	30	17.6	26	15.3	14	8.2
Middle-income countries	206	31.9	22	8	4	3	11	7	34	16.5	33	16	78	37.9
Low-income countries	269	41.7	23	13	10	3	9	2	46	17.1	45	16.7	68	25.3
Total	645	100	60	31	19	7	50	21	110	17.1	98	15.2	160	24.8

bMR, bivalent measles-rubella combined vaccine; mMe, monovalent measles vaccine; mRu, monovalent rubella vaccine; mMu, monovalent mumps vaccine; mV, monovalent varicella vaccine.

### 3.3 Association between vaccine cost-sharers and vaccination coverage

Among travelers traveling to low- and middle-income countries, there was a statistically significant difference between vaccinated and nonvaccinated travelers in the proportion of the cost of rabies vaccine borne by someone other than themselves, such as a company or school (*P* = 0.001). The *P*-values for hepatitis A and typhoid fever were 1.0 and 0.098, respectively, with no significant difference.

### 3.4 Adverse events

Fifteen patients (2.3%) experienced adverse events after vaccination. Adverse events included vasovagal reflex in five (33.3%) patients, redness and pain at the vaccination site in four (26.7%) patients, and prolonged bleeding due to venous puncture, the appearance of skin rash, fever, quadriceps pain (adult case), numbness of the left fifth finger, and cervical lymphadenopathy in one (6.7%) patient each. There were 10 (2.8%) adverse events after Havrix^®^, nine (3.6%) with Verorab^®^, four (3.4%) with Typhim Vi^®^, four (3.7%) with Boostrix^®^, two (3.3%) with Priorix^®^, and 0 (0%) with Mencevax^®^. Adverse events are summarized in Table 6.

**Table 6:** Frequency of adverse events after vaccination and their specific symptoms

Presence of adverse events	Present	15	2.3%
	None	617	95.7%
	No vaccination	13	2.0%
Symptom of adverse events	Vaso-vagal reflex	5	
	Erythema and pain*	4	
	Bleeding at the puncture site	1	
	Skin rash*	1	
	Fever (<37.5℃)*	1	
	Pain in the quadriceps*	1	
	Numbness in the fifth finger of the left hand*	1	
	Cervical lymphadenopathy*	1	
Frequency of adverse events	Havrix^#^	10	2.8%
	Verorab^#^	9	3.6%
	Typhim Vi^#^	4	3.4%
	Boostrix^#^	4	3.7%
	Mencevax^#^	0	0.0%
	Priorix^#^	2	3.3%
	Heptavax-II	1	0.5%
	JEBIK-V	3	1.9%
	ADSORBED TETANUS TOXOID	5	3.6%
	DRIED LIVE ATTENUATED MUMPS VACCINE	1	2.0%

^*^All symptoms resolved within a few days; ^#^Unapproved vaccines in Japan.

## 4 Discussion

The hepatitis A vaccine should be administered to citizens of developed countries who travel to middle- and low-income countries [2]. The vaccination rate has not reached 100%; however, this is due to a previous vaccination history or vaccination at another hospital. The results suggest that the hepatitis A vaccine is generally inoculated to those who travel to the country where it is needed and that clients of travel clinics and healthcare providers share the same consensus that the hepatitis A vaccine is, at least, mandatory for travelers to low- and middle-income countries.

The risk groups for hepatitis B include medical personnel, adventure travelers, Peace Corps volunteers, missionaries, military personnel, and medical travelers [2]. It is also believed that the administration of the hepatitis B vaccine should be considered for travelers who may come into contact with blood or bodily fluid secretions during travel, with potential for sexual contact or with a need for medical or dental procedures. In our experienced clients, there are not many within these categories, and it is our opinion that this is related to the low vaccination rate in our study. However, the hepatitis B vaccine has already been incorporated into the routine immunization program in Japan since 2016, and it is necessary to recommend it more aggressively as a catch-up vaccination.

Regarding meningococcal and pertussis-containing vaccines (particularly Tdap), vaccination rates are higher among travelers in high-income countries. This may be related to the fact that many students studying in developed countries in Europe and the United States are often required by their prospective schools to provide proof of Tdap and quadrivalent meningococcal conjugate vaccine (MCV4) vaccination. Meningococcal infection and whooping cough are not only prevalent in developed countries; however, meningococcal infections are also prevalent in middle-and low-income countries [3]. Therefore, travelers to low- and middle-income countries must be educated to be vaccinated against MCV4 in the future. Additionally, the MCV4 vaccine we are currently using is effective against four serotypes (A, C, Y, and W-135), however, not on serotype B. As the latter has been reported to be the most common serotype in Europe, the United States, and Latin America [4], a system must be established to provide vaccinations against serotype B in Japan.

Rabies vaccines are recommended for travelers visiting every country worldwide, except for rabies-free countries, including Japan, particularly for long-term visitors. However, many travelers do not wish to receive pre-exposure prophylaxis (PrEP) owing to the availability of post-exposure prophylaxis (PEP). Even if PEP is possible, PrEP remains important due to the severity of the disease when it occurs and the safety and availability of rabies immunoglobulin and should continue to be recommended. In 2018, the World Health Organization position paper [5] has been significantly revised to accept a reduction in the number of rabies vaccinations required to complete PrEP from three to two, making it possible to complete PrEP in a shorter period. Thus, more travelers will likely receive PrEP in the future.

The low vaccination coverage of typhoid fever and other travel vaccines among Japanese travelers has been a recurring problem in the past [6,7]. However, manufacturing approval of typhoid vaccine is not issued even now in Japan, and only individually imported typhoid vaccines can be used. Consequently, vaccination coverage among travelers to South Asia, where typhoid prevalence is high, is only approximately 50%. It is necessary to strongly recommend vaccination for people traveling to at-risk areas in the future. Another problem is that the typhoid vaccine is not approved for production in Japan, and there are very few medical facilities where it is available. Some travelers have received a complete set of travel vaccines except for the typhoid vaccine at another clinic and then visit us for the typhoid vaccine alone. As these cases show, the lack of approval for the production of typhoid vaccine in Japan reduces accessibility to the vaccine. We believe that approval for the production of typhoid vaccines in Japan is mandatory.

One of the reasons why travelers hesitate to receive the recommended vaccines is that they are expensive. It is true that highly recommended vaccines, such as hepatitis A, are administered regardless of the price of the vaccine. However, for the rabies vaccine, there was a significant difference in the breakdown of vaccine cost bearers between the vaccinated and non-vaccinated groups. The same trend was observed for the typhoid vaccine, although the difference was not significant (if the number of people enrolled in the study was twice as large as in this study, a significant difference was observed for the typhoid vaccine). Therefore, companies sending their employees abroad, schools sending their students abroad to study, and the Japanese government that encourages its citizens to be active abroad should actively work to subsidize or lower the cost of vaccines. There have been similar reports in the past [8], and our study reinforces the fact that vaccination coverage is associated with cost payers.

Since this was a retrospective study, we should emphasize that we were only able to assess adverse events occurring in the examination room just after the inoculation or unsolicited adverse event after returning home. The lower incidence of adverse events compared to previous reports [9,10,11,12,13,14] may be due to this reason. Nonetheless, no particularly serious adverse events were observed, and the frequency was not high, at approximately a few percentages. However, the vasovagal reflex was the most common event, which may lead to fainting or falling in some cases, suggesting the need to strictly monitor body posture during and after vaccination.

The following are the limiting factors of this study. First, this was a single-center study. We could observe only the trend of a single institution in this study, and it does not apply to the whole of Japan. Second, although we statistically examined the relationship between vaccination rates and cost bearers, there may be confounding factors. For example, the duration of planned travel may be longer when the vaccine is paid for by someone other than the patient. If the travel period is longer, the doctor will more strongly recommend rabies or typhoid vaccination. Third, it is up to the patient to decide whether or not symptoms after vaccination are reported as adverse events, except in the case of adverse events occurring immediately after vaccination. If swelling or pain occurs after vaccination, the patient may not call for help, while a patient who is concerned may call for help with even the slighter symptom.

## 5 Conclusion

At the travel clinic of Nara Medical University, the only vaccine that is adequately vaccinated for travelers in need is hepatitis A. Efforts should be made to provide rabies and typhoid vaccines more appropriately to travelers who need them. Regarding the rabies vaccine, it is expected that the vaccination rate can be improved by having the cost of the vaccine paid by someone other than the travelers themselves (such as the company sending the employee). Reducing the cost burden is also important for the spread of vaccines. The availability of typhoid vaccines is also a problem, as it has not yet been approved in Japan. Manufacturing approval for typhoid vaccines is necessary for Japan. Finally, in our study, the imported vaccines (Havrix^®^, Verorab^®^, Boostrix^®^, Priorix^®^, Typhim Vi^®^, Mencevax^®^) were used safely and well-tolerated.

## Abbreviations

GNI: gross national income
MCV4: quadrivalent meningococcal conjugate vaccine
PEP: post-exposure prophylaxis
PrEP: pre-exposure prophylaxis
